# Supplementary material for: In Silico Screening of Bioactive Compounds of Representative Seaweeds to Inhibit SARS-CoV-2 ACE2-Bound Omicron B.1.1.529 Spike Protein Trimer
Source: Mar Drugs. 2022 Feb 17;20(2):148. doi: 10.3390/md20020148 (PMC8877529; doi:10.3390/md20020148)
Supplement: Supplementary file 1 [file marinedrugs-20-00148-s001.zip › marinedrugs-1602736-supplementary.pdf]

Supplementary File

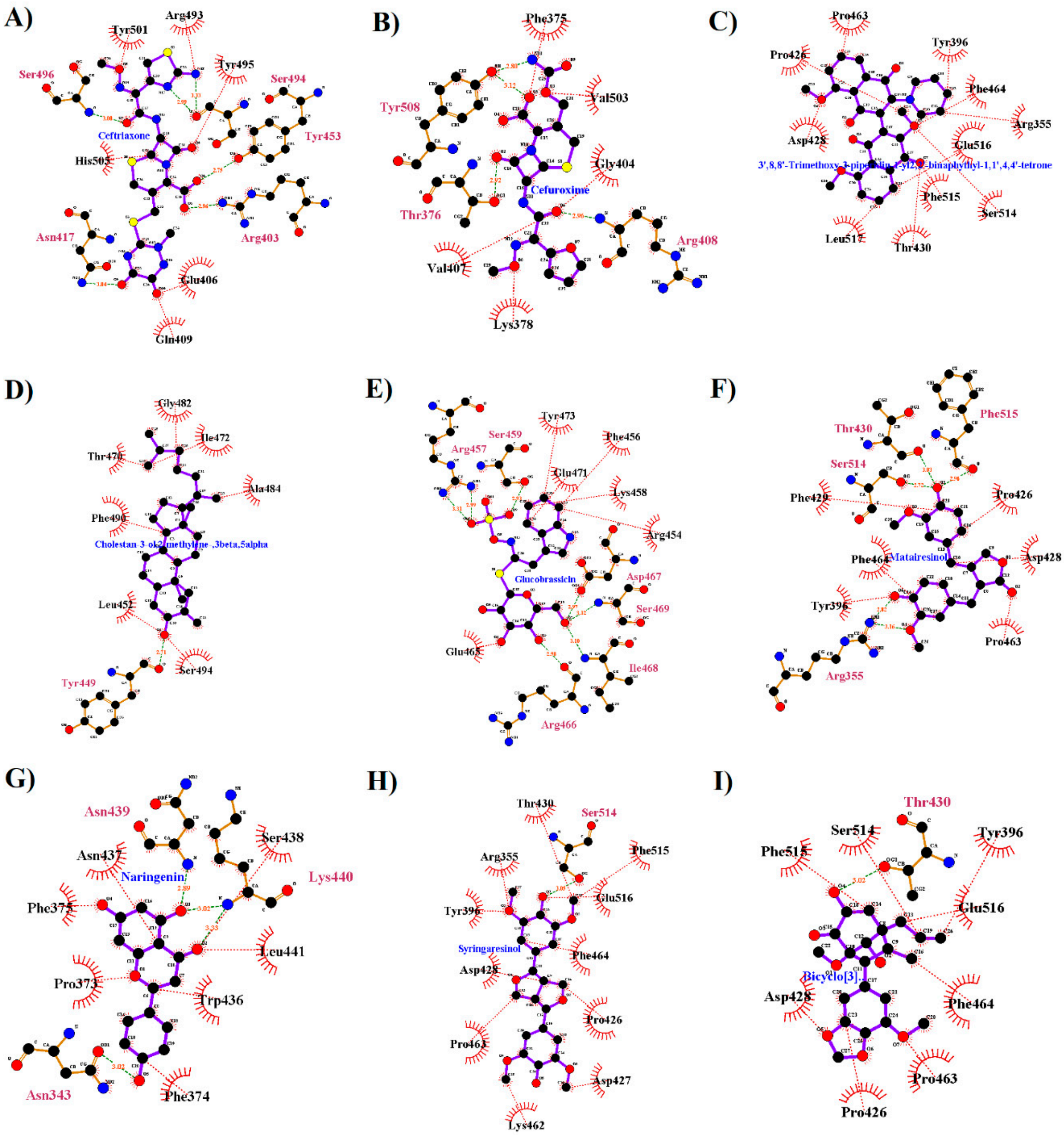

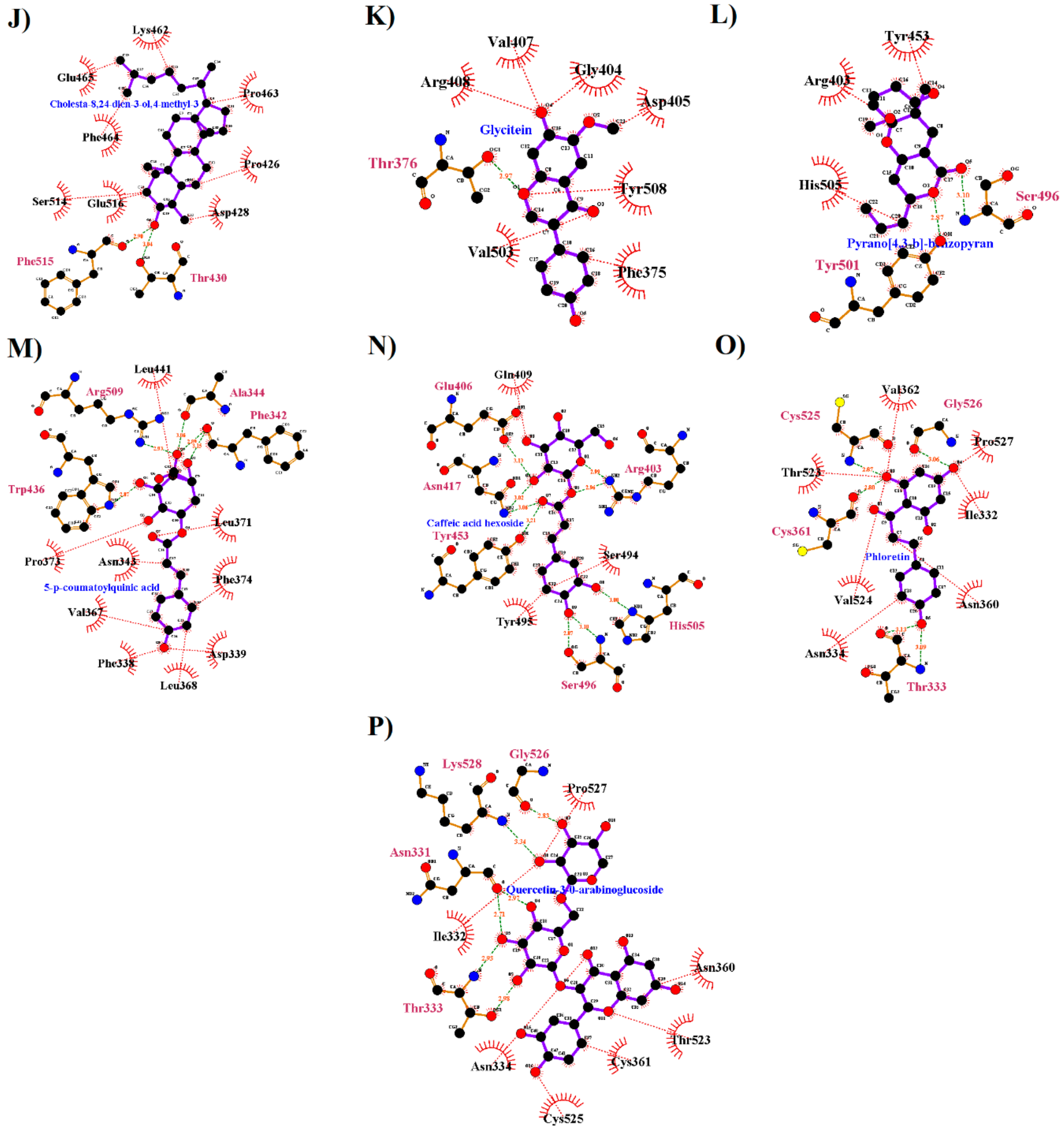

Figure S1: The Ligplot interaction between the analyzed compounds and the RBD of omicron B.1.1.529 spike protein with the binding affinity of ≤ -6.0 kcal/mol. **A)** Ceftriaxone-RBD, **B)** Cefuroxime, **C)** 3',8,8'-Trimethoxy-3-piperi-din-1-yl2,2'-binaphthyl-1,1',4,4'-tetrone, **D)** Cholestan-3-ol, 2-methylene-, (3beta, 5 alpha), **E)** Glucobrassicin, **F)** Matairesinol, **G)** Naringenin, **H)** Syringaresinol, **I)** Bicyclo[3.2.1]oct-3-en-2-one, 3,8-dihydroxy-1-methoxy-7-(7-methoxy-1,3-benzodioxol-5-yl)-6-methyl-5, **J)** Cholesta-8,24-dien-3-ol, 4-methyl-, (3.beta.,4.alpha.)-, **K)** Glycitein, **L)**Pyrano [4,3-b] benzopy-ran-1,9-dione, 5a-methoxy-9a-methyl-3-(1-propenyl) perhydro, **M)** 5-p-coumaroylquinic acid, **N)** Caffeic acid hexoside, **O)** Phloretin, **P)** Querce-tin-3-O-arabinoglucoside.

Table S1: The list of seaweeds and compounds utilized in the current study

S. No.	Seaweeds and Phytocompounds	Binding affinity (kcal/mol)	RMSD (Å)
Ulva fasciata [1]			
1.	Azelaic acid	-4.5	2.063
2.	n-Pentadecanoic acid	-4.5	1.426
3.	Hexahydrofarnesyl acetone	-5.0	1.555
4.	Palmitic acid	-4.0	3.37
5.	Palmitic acid ethyl ester	-3.7	1.81
Corallina officinalis [2]			
6.	n-Nonadecane	-4.4	12.43
7.	Chloroacetic acid, octadecyl ester,	-4.5	1.803
8.	Cholestan-3-ol, 2-methylene-, (3beta, 5 alpha)	-6.0	3.074
9.	2-Methylhexadecan-1-ol	-3.5	1.88
10.	1-Docosene	-4.3	1.957
11.	1,2,3- Propanetricarboxylic acid, 2-(acetyloxy)-,tributyl ester	-4.5	1.95
12.	1,1-Dimethyltetradecyl hydrosulfide	-3.7	24.717
13.	1-Eicosanol	-4.7	3.579
14.	1,54-Dibromotetra Pentacontane	-1.0	29.281
15.	3',8,8'-Trimethoxy-3-piperidin-1-yl2,2'-binaphthyl-1,1',4,4'-tetrone	-6.9	1.897
16.	(17E)-17-Pentatriacontene	-1.0	0.165
17.	2-Octadecoxyethanol	-3.7	5.639
Colpomenia sinuosa [2]			
18.	n-Tetradecanoic acid	-4.3	4.283
19.	Tetradecanoic acid, ethyl ester	-4.3	4.243
20.	6,10,14-Trimethylpentadecan-2-one	-4.3	4.454
21.	n-Hexadecanoic acid	-4.3	22.956
22.	n-Tridecanoic acid, ethyl ester	-4.6	1.701
23.	n-Pentadecanoic acid, ethyl ester	-4.2	1.93
24.	Ethyl (9Z,11E)-9,11-octadecadienoate	-4.0	19.205
25.	Ethyl n-heptadecanoate	-3.7	2.473
26.	Bis (2-ethylhexyl) 1,2-benzenedicarboxylate	-5.2	1.976
27.	Ethyl n-hexadecanoate	-3.9	4.736
Sargassum wightii [3]			
28.	Phloretin	-6.3	0.061
29.	Heptadecanoic acid	-4.1	1.658
30.	Oleic acid	-4.4	1.747
31.	5-p-coumaroylquinic acid	-6.0	4.96
32.	Quercetin-3-O-arabinpyranoside	-6.1	2.248
33.	Caffeic acid hexoside	-6.4	2.82
34.	Squalene	-3.6	19.813
Gracilaria corticate [4]			

35.	Oxirane, decyl-	-3.9	12.931
36.	n-hexadecanoic acid	-4.4	1.478
37.	Eicosanoic acid	-4.6	6.426
38.	Nonanoic acid	-4.2	3.19
39.	Oleic acid	-4.6	6.711
40.	Pentadecanoic acid	-4.3	6.226
41.	Bicyclo[3.2.1]oct-3-en-2-one, 3,8-dihydroxy-1-methoxy-7-(7-methoxy-1,3-benzodioxol-5-yl)-6-methyl-5	-6.3	20.322
42.	N-(5-chloro-2-hydroxyphenyl) dodecanamide	-5.3	1.674
43.	Cholesta-8,24-dien-3-ol, 4-methyl-, (3.beta.,4.alpha.)-	-6.8	1.544
44.	Undecane, 2-methyl-	-3.4	1.411
45.	Hexadecanal	-4.3	2.192
46.	Triacontanoic acid, methyl ester	-0.9	29.217
Caulerpa racemosa [5]			
47.	6-Hydroxy-4, 4,7atrimethyl-5, 6, 7,7atetrahydro-1-benzofuran-2(4H)-one	-5.5	2.004
48.	Hexadecanoic acid, methyl ester	-4.2	5.929
49.	n-Hexadecanoic acid	-3.9	39.871
50.	Palmitic anhydride	-4.8	28.901
51.	Rhodovibrin	-0.9	32.477
52.	Phytol	-5.2	2.959
53.	9,12-Octadecadienoic acid (Z, Z)	-4.6	1.291
54.	Oleic acid	-4.9	1.907
55.	Methyclothiazide	-5.1	2.546
56.	Glycidyl palmitate	-4.1	1.531
57.	2-cis,cis-9,12-Octadecadienyloxy ethanol	-3.7	13.714
58.	1H-Indene, 1-hexadecyl-2,3-dihydro	-5.5	3.585
59.	Glycerol 1-palmitate	-3.6	1.504
60.	1H-cyclopropal	-	-
61.	Diisooctyl phthalate	-4.6	1.936
62.	17-Octadecynoic acid, TMS derivative	-	-
63.	Linoleic acid ethyl ester	-3.7	3.064
64.	Glucobrassicin	-6.8	1.521
65.	Oleic acid, eicosyl ester	-4.9	1.907
66.	5(α)Pregane3α,20αdiol	-	-
67.	6-Hydroxy-4, 4,7atrimethyl-5, 6, 7,7atetrahydro-1-benzofuran-2(4H)-one	-5.5	2.004
Padina boergesenii [5]			
68.	Glycitein	-6.1	11.668
69.	Matairesinol	-6.0	1.707
70.	9-Hexadecenoic acid, methyl ester (Z)	-	-
71.	Hexadecanoic acid, methyl ester	-4.1	1.82
72.	n-Hexadecanoic acid	-4.5	5.689
73.	Pyrano [4,3-b] benzopyran-1,9-dione, 5amethoxy-9amethyl-3-(1-propenyl) perhydro	-6.1	29.617
74.	Butanoic acid, 4-chloro	-4.9	2.083

75.	Arachidonic acid	-4.9	1.578
76.	Naringenin		
77.	11,14-Octadecadienoic acid, methyl ester	-4.3	1.409
78.	9-Octadecenoic acid (Z)-, methyl ester	-3.9	4.343
79.	Phytol	-4.1	2.174
80.	13-Heptadecyn-1-ol	-4.4	1.775
81.	cis-Vaccenic acid	-4.0	1.437
82.	5,8,11,14 -Eicosatetraenoic acid, methyl ester, (all-Z)	-	-
83.	1H-Cyclopropa [3,4] benz [1,2-e] azulene4a,5,7b,9,9a (1aH)	-	-
84.	Stearic acid, 3-(octadecyloxy) propyl	-	-
85.	Glycidyl oleate	-4.5	3.938
86.	(1S)-1-Hexadecyl-2,3-dihydro-1Hindene	-5.1	4.57
87.	2-Oleoylglycerol, 2TMS derivative	-	-
88.	Cyclohexane, 1,1-dodecylidenebis [4-methyl]	-5.3	1.154
89.	Glucobrassicin	-6.8	1.521
Gracilaria edulis [6]			
90.	Eugenol	-4.5	1.213
91.	Nonane	-3.8	1.844
92.	Undecane	-4.1	1.277
93.	Hept-2-ene, 2,4,4,6-tetramethyl	-4.3	0.927
94.	Sulfurous acid	-3.3	1.244
95.	Phthalic acid	-4.8	1.595
96.	1,2-Propanediol	-3.6	1.388

References

1. Rodeiro, I.; Olguín, S.; Santes, R.; Herrera, J.A.; Pérez, C.L.; Mangas, R.; Hernández, Y.; Fernández, G.; Hernández, I.; Hernández-Ojeda, S.; Camacho-Carranza, R.; Valencia-Olvera, A.; Espinosa-Aguirre, J.J. Gas chromatography-mass spectrometry analysis of *Ulva fasciata* (Green Seaweed) extract and evaluation of its cytoprotective and antigenotoxic effects. *Evid. Based. Complement. Alternat. Med.* **2015**, *2015*, 520598.
2. Hassan, S.; Shobier, A. GC/MS identification and applications of bioactive seaweed extracts from Mediterranean coast of Egypt. Egypt. *J. Aquat. Biol. Fish.* **2018**, *22*, 1-21.
3. Deepak, P.; Sowmiya, R.; Kamaraj, C.; Josebin, M.; Aiswarya, D.; Govindasamy, B.; Amutha, V.; Perumal, P. GC-MS profiling, chemical characterization, antioxidant, α-amylase and α-glucosidase inhibition of selected seaweeds from southeast coast of India, in vitro approach. *J. drug deliv. ther.* **2018**, *8*, 29-40.
4. Kumari, P.; Reddy, C.R.; Jha, B. Comparative evaluation and selection of a method for lipid and fatty acid extraction from macroalgae. *Anal. Biochem.* **2011**, *415*, 134-44.
5. Ragunath, C.; Kumar, Y.A.S.; Kanivalan, I.; Radhakrishnan, S. Phytochemical screening and GC-MS analysis of bioactive constituents in the methanolic extract of

caulerpa racemosa (Forssk.) j. agardh and *padina boergesenii* allender & kraft. Curr. *Appl. Sci. Technol*. **2020**, *20*, 380-393.

6. Arulkumar A, Rosemary T, Paramasivam S, Rajendran R. Phytochemical composition, in vitro antioxidant, antibacterial potential and GC-MS analysis of red seaweeds (Gracilaria corticata and Gracilaria edulis) from Palk Bay, India. Biocatal. *Agric. Biotechnol.* **2018**, *15,* 63-71.
